# Carbonic anhydrase 9 (CA9) expression in non-small-cell lung cancer: correlation with regulatory FOXP3+T-cell tumour stroma infiltration

**DOI:** 10.1038/s41416-020-0756-3

**Published:** 2020-02-18

**Authors:** Alexandra Giatromanolaki, Adrian L. Harris, Alison H. Banham, Constantinos A. Contrafouris, Michael I. Koukourakis

**Affiliations:** 1 Department of Pathology, University Hospital of Alexandroupolis, Democritus University of Thrace, Alexandroupolis, Greece; 2 Cancer Research UK, Molecular Oncology Laboratories, Weatherall Institute of Molecular Medicine, University of Oxford, Oxford, UK; 3 Nuffield Division of Clinical Laboratory Sciences, Radcliffe Department of Medicine, John Radcliffe Hospital, Oxford, OX3 9DU UK; 4 1st Cardiac Surgery Department, Onassis Cardiac Surgery Center, 17674 Athens, Greece; 5 Department of Radiotherapy/Oncology, University Hospital of Alexandroupolis, Democritus University of Thrace, Alexandroupolis, Greece

**Keywords:** Translational research, Cancer microenvironment

## Abstract

**Background:**

Low pH suppresses the proliferation and cytotoxic activity of CD8+ cytotoxic and natural killer lymphocytes. The hypoxia-regulated transmembrane protein, carbonic anhydrase CA9, converts carbon dioxide produced by the Krebs cycle to bicarbonate and protons that acidify the extracellular milieu. We examined whether CA9 is also involved in intratumoural immunosuppression pathways.

**Methods:**

A series of 98 tissue samples of primary non-small-cell lung carcinomas (NSCLC) from patients treated with surgery were analysed for the expression of CA9 and programmed-death ligand PD-L1 by cancer cells, and of FOXP3 by tumour-infiltrating lymphocytes (TILs).

**Results:**

There was no direct association of CA9 with PD-L1 expression or the density of TILs in the tumour stroma, but CA9 was directly related to the extent of FOXP3+ TIL density (*p* = 0.008). Double-stratification survival analysis showed that patients with high CA9 expression and low TIL score had significantly poorer survival compared with all other groups (*p* < 0.04). In a multivariate analysis stage (*p* < 0.0001, HR 1.95, 95% CI: 1.3–2.7), TIL score (*p* = 0.05, HR 0.55, 95% CI: 0.2–1.0) was an independent prognostic variable of death events. CA9 expression by cancer cells is associated significantly with FOXP3+ regulatory T-cell abundance in the tumour stroma of NSCLC.

**Conclusion:**

The study provides a basis for testing CA9 as a marker of resistance to immune-checkpoint inhibitors and as a therapeutic target to enhance the efficacy of immunotherapy.

## Background

Immunotherapy with immune-checkpoint inhibitors has revolutionised the therapy of metastatic disease for several human malignancies, including non-small-cell lung cancer (NSCLC). Anti-PD-1/PD-L1 monoclonal antibodies, alone or in combination with chemotherapy, have been approved for the treatment of advanced NSCLC, following randomised trials that confirmed the improvement of the overall survival and a better toxicity profile compared with chemotherapy.^1^ Nevertheless, despite the documented prolongation of life, the response rates to immunotherapy are less than 25% in previously untreated patients, which increases to 35% when tumours extensively express PD-L1.^2^ Moreover, responders will eventually relapse. Lack of PD-L1 expression, co-expression of other disrupting immune-checkpoint co-inhibitory molecules or impaired antigen presentation machinery by cancer cells contributes to the failure of anti-PD-L1 therapy to control the disease.^3^

The tumour microenvironment is also strongly implicated in tumour evasion from immune surveillance. Intratumoural hypoxia, through HIF1α activation, promotes the secretion of cytokines (e.g. IL-6 and IL-8) involved in the differentiation of myeloid dendritic cells towards an immunosuppressive phenotype.^4^ Hypoxia also promotes HIF1α-dependent overexpression of CD47 or PD-L1 immune-checkpoint inhibitory molecules by cancer cells or tumour-infiltrating immune cells.^5,6^ Enhanced production of lactate, through anaerobic glycolysis and subsequent extracellular transport of lactate and protons by monocarboxylate transporters, is also a major pathway inducing acidosis in the tumour microenvironment.^7^ Another pathway that promotes intratumoural acidosis involves glutamine metabolism that feeds the Krebs cycle with glutamate and α-ketoglutarate, sustaining oxidative metabolism under hypoxia, producing CO_2_.^8^ CO_2_ is hydrated by the transmembrane protein carbonic anhydrase 9 (CA9) and converted to bicarbonate and protons that acidify the extracellular milieu.^9^ In addition, hypoxia-induced bicarbonate transporters import HCO3^−^ ions, which combine with intracellular acid to produce CO_2_ that diffuses out of the cell, a gradient maintained by CA9.^10^ Low pH markedly suppresses the proliferation and cytotoxic activity of CD8+ cytotoxic and natural killer lymphocytes.^11–13^

In a previous study, we showed that CA9 is strongly expressed in about a third of NSCLC, and is correlated with angiogenic pathways and poor prognosis in operable disease.^14^ In this study, we provide evidence that CA9 is also involved in intratumoural immunosuppression pathways.

## Methods

A series of 98 tissue samples of primary non-small-cell lung carcinomas (NSCLC), from patients treated with surgery, were retrieved from the archives of Pathology, Democritus University of Thrace. Patient and disease characteristics are shown in Table 1.

**Table 1:** Patients’ characteristics (*n* = 98).

*Age*	
Median	68
Range	32–81
*Sex*	
Male	86
Female	12
*Stage*	
I	46
II	22
III	30
IV	0
*Tumour cell type*	
Squamous cell carcinoma	58
Adenocarcinoma	22
Large-cell carcinoma	18
*Histology grade*	
Squamous cell carcinoma	
1	45
2	8
3	5
Adenocarcinoma	
1	7
2	12
3	3
*Follow-up in months*	
Median	46
Range	26–112

### Immunohistochemistry

Immunohistochemistry was performed on 3-μm-thick formalin-fixed paraffin-embedded tissue sections. Details in the methodology applied have been previously reported.^15^ The Dako EnVision FLEX kit was used. The well-validated rabbit monoclonal PD-L1 antibody (clone CAL10, Biocare Medical, CA, USA) was used at a dilution of 1:100 and 60-min incubation at room temperature.^16^ For FOXP3^+^ Treg detection, we used ‘in house’ undiluted hybridoma supernatant from the well-validated murine monoclonal antibody 236A/E7 that is widely used for FOXP3 detection in routinely fixed tissues.^17^ For CA9 detection, the mouse monoclonal M75 antibody was used, at dilution 1/200 and 60-min incubation.

In each run of immunohistochemical staining, a tissue section from normal gastric or gallbladder tissue (Supplementary Fig. 1s) was included as a CA9-positive control. Normal lung tissue was used as a negative control. Tissue sections from a reactive (inflammatory) lymph node with extensive presence of FOXP3+ lymphocytes were used as a positive control in each immunohistochemistry run. FOXP3-positive, when present, and negative lymphocytes function also as a positive or negative internal control, respectively, in each slide.

The percentage of cancer cells with membrane CA9 and with strong membrane (with or without cytoplasmic) PD-L1 reactivity was assessed in all available optical fields at ×200 magnification, and the mean values were used to score each case. Cases were grouped as negative (lack of expression), as of low (limited expression in 1–9% of cells), medium (10–49% of cells) and high expression (>50 of cells).

### Assessment of TIL score and FOXP3+ TIL (FIL) score

Tumour-infiltrating lymphocytes (TILs) were assessed in the FOXP3-immunostained slides. The number of haematoxylin-stained TILs was assessed in the stroma (not in tumour nests), in the entire tissue section and in ×40 optical fields, and the mean value defined the final score for each case. Lymphocytes were recognised by morphological criteria, and there was no T-cell-specific staining applied. All mononuclear cells (including lymphocytes and plasma cells) were taken into account, while polymorphonuclear leukocytes and macrophages are excluded. Four different *TIL-score* categories were initially defined subjectively for scoring (minimal, low, medium and high). Counting the absolute numbers of lymphocytes per optical field, it was noted that *TIL-score* 1 (or minimal) defined cases with 1–10 lymphocytes/o.f., 2 (or low) 10–70 lymphocytes/o.f., 3 (or medium) 70–150 lymphocytes/o.f. and 4 (or high) >150 lymphocytes/o.f.

The percentage of FOXP3-expressing lymphocytes among the TILs present in the tumour stroma was assessed in the entire tissue section, in ×40 optical fields, and the mean score was used for each case. This score provides only the % of TILs expressing FOXP3, and does not reveal the extent of FOXP3 lymphocytic infiltration in the tissue, which also depends upon the extent of TIL presence. The *FIL score* was therefore assessed as the product of ‘*TIL-score'*  ×  ‘*% FOXP3+* *TILs'*.

### Statistical analysis

Statistical analysis was performed using the GraphPad Prism 5.0 package. The chi-square or Fisher’s exact *t* test was used to compare categorical variables as appropriate. Kaplan–Meier survival curves were used to assess the impact of assessed variables on the disease-specific overall survival of patients. Α Cox’s proportional hazard regression model using backward elimination was applied to assess the effect of the parameters on the death events. These included the CA9 expression (low/negative vs. medium/high), stage (1 vs. 2 vs. 3), the TIL score (1,2 vs. 3,4) and the FIL score (0 vs. positive). A *p*-value of <0.05 was used for significance.

## Results

### Expression of CA9

**Fig. 1:**
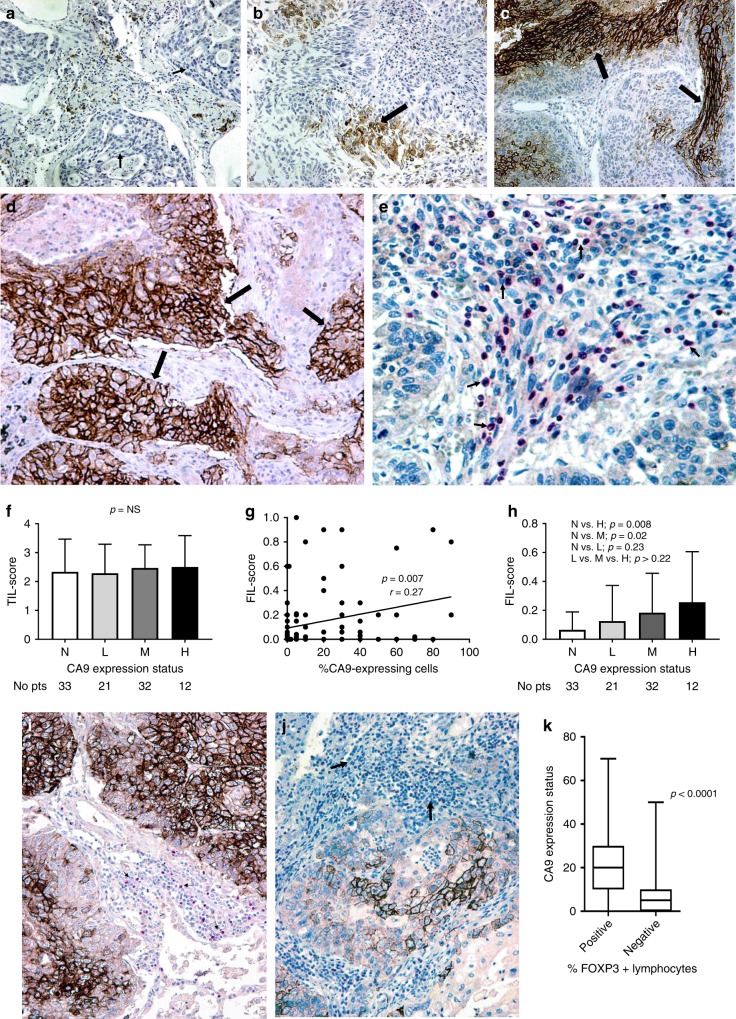
Immunohistochemical figures and figures showin associations between CA9 expression and lymphocyte-related parameters. **a** Immunohistochemical image showing lack (grouped as negative) of cancer cell CA9 reactivity (arrows) in a squamous cell lung cancer (magnification ×20). **b** Immunohistochemical image showing sporadic (grouped as low) staining (arrows) of cancer cell CA9 reactivity in a squamous cell lung cancer (magnification ×20). **c** Immunohistochemical image showing CA9 staining (arrows) in less than 50% of cancer cells (grouped as medium) in a squamous cell lung cancer (magnification ×20). **d** Immunohistochemical image showing extensive membrane CA9 reactivity (arrows) in >50% of cancer cells of a squamous cell lung cancer (magnification ×20). **e** Immunohistochemical image showing intense infiltration of tumour stroma by FOXP3+ lymphocytes (arrows), in a squamous cell lung cancer (magnification ×40). **f** TIL score according to the four CA9 expression categories (absence/N vs. low/L vs. medium/M vs. high/H expression). Bars show standard deviation. **g** Linear regression analysis of the percentage of CA9-expressing cancer cells and the FIL score. **h** FIL score according to the four CA9 expression categories (absence/N vs. low/L vs. medium/M vs. high/H expression). Bars show standard deviation. **i** Double immunostaining for CA9 and FOXP3 in a squamous cell lung cancer extensively expressing CA9; arrows show FOXP3+ lymphocytes (magnification ×20). **j** Double immunostaining for CA9 and FOXP3+ in a squamous cell lung cancer showing poor expression of CA9 in cancer cells, and lack of expression of FOXP3 by tumour-infiltrating lymphocytes (arrows) (magnification ×20). **k** Percentage of FOXP3+ infiltrating lymphocytes in the stroma according to the extent of expression of CA9 adjacent to the stroma cancer cells, assessed in 20 selected double CA9/FOXP3-immunostained tissue slides. Bars show standard deviation (magnification ×20).

Membrane expression of CA9 was noted in a varying percentage of cancer cells, ranging from 0 to 90% (median 5%, mean 16.7%). No expression (0%) was noted in 33/98, low expression (1–9%) in 21/33, medium expression (10–49%) in 32/98 and high (>50) in 12/98 cases. Figure 1a–d shows representative immunohistochemical images from these four staining patterns.

There was no association between stage, histology, tumour grade and CA9 expression. Regarding the association of CA9 with PD-L1 expression, there was no significant association, as 14/44 (31.8%) cases with medium/high CA9 expression had medium/high PD-L1 expression vs. 9/54 (18.5%) of cases with negative/low CA9 expression (*p* = 0.09).

### Lymphocytic infiltration

Out of 98 cases, 20 (20.5%) had a TIL score equal to 1, 36 (36.7%) a score of 2, 26 (26.5%) a score of 3 and 16 (16.3%) a score of 4. The percentage of TILs with FOXP3 expression ranged from 0 to 50% (median 1%, mean 6% and 75th percentile 10%). Figure 1e shows a case with extensive presence of FOXP3+ lymphocytes in the tumour stroma. The *FIL score* (‘*TIL-score’*  × ‘%*FOXP3+* *cells’*) ranged from 0 to 1 (median 0.02). Out of 98 cases, 47 (47.9%) had a *FIL score* equal to 0 (zero ‘0’), 35 (35.7%) had a score of 0.02–0.2 and 16 (16.3%) a score of 0.21–1.

### Correlation of CA9 with lymphocyte parameters

There was no significant difference between the *TIL scores* obtained in the four groups defined by CA9 expression levels (Fig. 1f). Linear regression analysis revealed a significant direct association between CA9 expression and FIL score (*p* = 0.007, *r* = 0.27, Fig. 1g). High CA9 expression had a mean FIL score of 0.25 vs. 0.06 in tumours lacking CA9 expression (*p* = 0.008, Fig. 1h).

We further performed double staining for CA9 and FOXP3 on 20 selected cases that expressed CA9 in 30–60% of the cancer cell population, containing areas with positive expression and lack of expression. In these slides, we assessed the percentage of FOXP3+ lymphocytes in the stroma adjacent to CA9-positive and -negative areas (a total of paired 60 areas were evaluated). This was significantly higher in the stroma-adjacent cancer cell areas with strong CA9 expression (median value 20% vs. 5%, mean value 22.3% vs. 9.2%, *p* < 0.00001) (Fig. 1i–k).

### Survival analysis

**Fig. 2:**
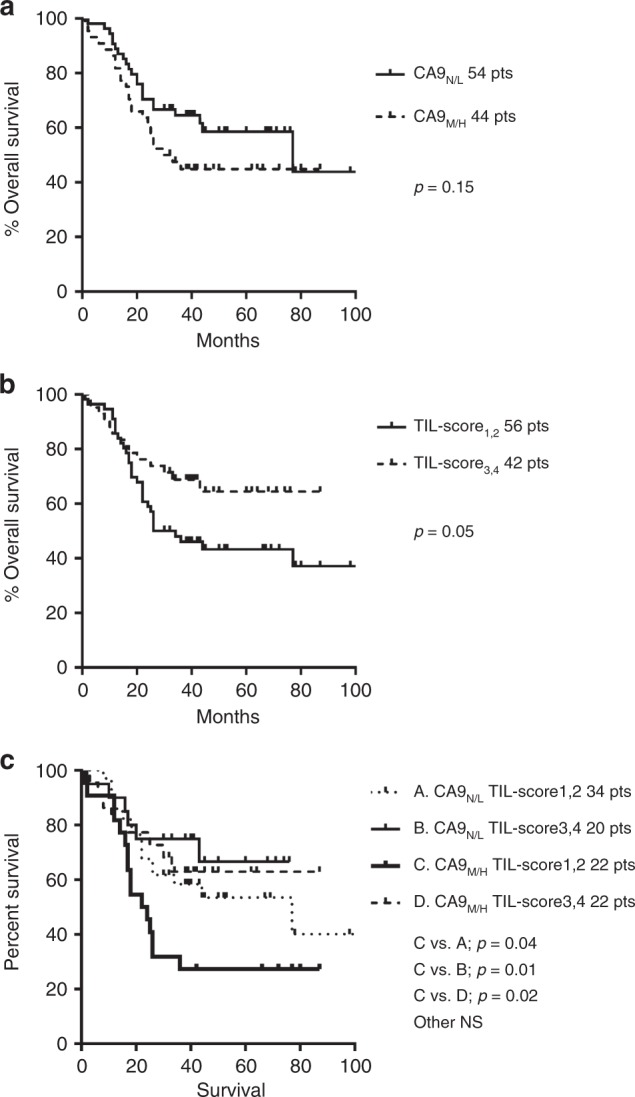
Kaplan–Meier overall (disease-specific) survival curves. **a** Stratified for CA9 expression (negative—N/low—L vs. medium—M/high—H), **b** stratified for TIL score (low 1,2 vs. high 3,4) and **c** double stratification for CA9 and TIL score.

The absence of, or low, CA9 expression defined favourable survival curves compared with medium-/high-expression groups, but the difference did not reach statistical significance (Fig. 2a, *p* = 0.15). Intense infiltration of the tumour stroma by TILs (*TIL score* 3,4) was significantly related to a better prognosis (Fig. 2b, *p* = 0.05). Double stratification, according to *TIL score* and CA9 expression, showed that patients with medium/high CA9 expression and low TIL score (1,2) had significantly poorer survival compared with all other groups (Fig. 2c, *p* < 0.04).

**Fig. 3:**
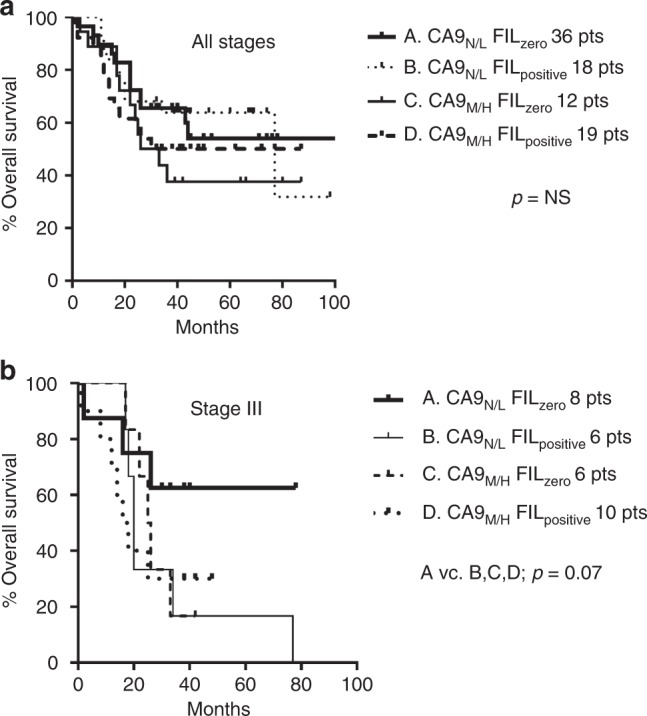
Figures of survival analysis. Kaplan–Meier overall (disease-specific) survival curves stratified for CA9 expression (negative—N/low—L vs. medium—M/high—H) and FIL score (zero vs. positive) in all cases (**a**) and in stage III cases (**b**).

For categorical analysis, the *FIL score* was divided into two categories (0 vs. > 0), thus zero vs. positive. Overall, there was no association of *FIL score* with overall survival (*p* = 0.91). Double-stratification analysis according to CA9 expression (negative/low vs. medium/high) and FIL score (zero vs. positive) did not show groups of patients with different prognosis (Fig. 3a). Despite the low number of cases, a similar double-stratification analysis in stage III patients showed that patients who lacked CA9 expression and FOXP3+ lymphocytes had a better prognosis, although of marginal significance (*p* = 0.07, Fig. 3b).

In a multivariate analysis, including stage, histology, TIL score, FIL score and CA9, stage (*p* < 0.0001, HR 1.95, 95% CI: 1.3–2.7) and TIL score (*p* = 0.05, HR 0.55, 95% CI: 0.2–1.0) were independent prognostic variables of death events.

## Discussion

Cytotoxic T-cell activity, mainly mediated by activated CD8+ T cells (Tcyt), is important in the maintenance of an immunological equilibrium that impedes tumour growth.^18^ Shifting this equilibrium towards tumour elimination, by targeting immune co-inhibitory molecules that suppress Tcyt activity, is under thorough clinical and laboratory investigation. Nevertheless, Tcyt activity is also regulated by a subset of CD4 + /CD25 + regulatory T cells that express FOXP3 (regulatory T cells or Tregs). FOXP3 is essential for the development of natural CD4^+/^CD25^+^ Tregs in humans,^19^ although it also characterises CD4 + Tregs independently of CD25 expression.^20^ Under normal conditions, circulating Tregs represent 5% of the total CD4+ lymphocytic population, while their presence increases in cancer patients.^21^

Intense FOXP3+ lymphocyte infiltration relates to poor prognosis,^22^ and indeed this has been documented in patients with NSCLC.^23–28^ In a recent study, we found that tumour infiltration by FOXP3+ Tregs occurs early in the development of NSCLC, and adversely affects the post-operative outcome.^15^ Tregs may compromise the activity of immune-checkpoint inhibitors, so that assessment of their presence could be a useful marker to guide immunotherapy. Therapeutic interventions targeting this very T-cell population may also prove to be important to improve survival of patients. It seems, therefore, important to study the reasons for Treg expansion and tumour infiltration in cancer patients, aiming to devise methods to remove this principal obstacle, and enhance the activity of modern immunotherapy.

A hypoxic tumour microenvironment promotes local immunosuppression through many biological pathways, including lactate release by cancer cells, acidosis, production of immunosuppressive molecules like adenosine and HIF1α-driven overexpression of checkpoint co-inhibitory molecules, like PD-L1 and CD47.^5,6,29^ HIF1α is a transcriptional regulator of CA9 and of lactate dehydrogenase A.^30,31^ In a previous study, we showed that LDHA is directly linked with PD-L1 expression in lung cancer cells, a relation that can be explained by the hypoxic environment of LDHA-expressing tumours.^32^ In this study, we also noted a trend for CA9-overexpressing tumours to have higher levels of PD-L1 expression. These data confirm at the clinical level that a hypoxic tumour microenvironment promotes PD-L1 expression and immune-checkpoint inhibition.

Aside to this direct effect of hypoxia on checkpoint inhibitory molecule expression, several studies suggest that cytotoxic T-cell proliferation and activity are severely compromised under the acidic conditions of the tumour microenvironment.^10,11^ In addition, chemokines produced by cancer cells under acidic and hypoxic conditions, induce chemotaxis of Tregs.^33^ HIF activation also seems to induce differentiation of CD4+ cells to FOXP3-expressing Tregs, either by direct binding to the FOXP3 regulatory region or by induction of TGFβ.^34,35^

Carbonic anhydrase 9 is a HIF1α-regulated downstream gene, and is involved in the acidification of the extracellular matrix by hydrating carbon dioxide to produce bicarbonate and protons.^9^ In this study, we provide evidence that CA9 expression by cancer cells is directly related to intense infiltration of the tumour stroma by FOXP3+ Tregs in NSCLC. A similar finding has been reported by Yan et al.^36^ in breast cancer, who also reported that hypoxia upregulated CXCR4 on Tregs. This association can be explained by the fact that CA9 is a hypoxia-regulated gene, directly regulated by HIF1α, so that the correlation found between CA9 expression and FOXP3 + TILs may be an indirect result of the hypoxic tumour environment and not a direct effect of acidity conferred by the carbonic acid released. Whether acidity per se can directly promote Treg chemotaxis, or can, similarly to hypoxia, promote differentiation of CD4+ T cells to FOXP3+ Tregs, remains obscure.

In any case, CA9, appearing to be a marker of tumour aggressiveness by reflecting active hypoxia pathways, is also a marker of local immunosuppression and Treg accumulation. Indeed, tumours with low CA9 expression and intense TIL presence in the tumour stroma defined a group of patients with optimal prognosis. Of interest, and despite the low number of patients analysed with stage III disease, lack of CA9 expression and absence of FOXP3+ lymphocytic infiltration also defined a group with better prognosis.

It is concluded that CA9 is a marker strongly associated with FOXP3+ regulatory T-cell abundance in the tumour environment of NSCLC. Whether CA9-positive NSCLCs are resistant to immune-checkpoint inhibitors should be sought in translational studies. Such patients would, eventually, benefit from a combination of immunotherapy with pharmaceutical agents that neutralise extracellular tumour acidity, such as CA9 and bicarbonate transport inhibitors.^37–40^

## Supplementary information

LEGEND of Figure 1s

Figure 1s

## Data Availability

All data reported in the study are available in our departments.
