# Supplementary material for: Carbonic anhydrase 9 (CA9) expression in non-small-cell lung cancer: correlation with regulatory FOXP3+T-cell tumour stroma infiltration
Source: Br J Cancer. 2020 Feb 18;122(8):1205–10. doi: 10.1038/s41416-020-0756-3 (PMC7156529; doi:10.1038/s41416-020-0756-3)
Supplement: Supplementary file 1 — LEGEND of Figure 1s [file 41416_2020_756_MOESM1_ESM.docx]

**Figure 1s (supplemental)**

Immunohistochemical images of tissues used as controls in each run of immunohistochemistry: (a, b) Reactive lymph node stained for FOXP3 showing red stained lymphocytes at x20 and x40 magnification, respectively, used as a positive control for FOXP3 immunohistochemistry; (c,d) Normal gastric and gallbladder tissue, respectively, showing intense CA9 membrane staining of the glandular epithelium, used as a positive control for CA9 immunohistochemistry (e) Normal lung tissue showing bronchial epithelium and seromucinous glands with lack of CA9 staining, used as a negative control for CA9 immunohistochemistry.
