# Supplementary figures and images for: Carbonic anhydrase 9 (CA9) expression in non-small-cell lung cancer: correlation with regulatory FOXP3+T-cell tumour stroma infiltration
Source: Br J Cancer. 2020 Feb 18;122(8):1205–10. doi: 10.1038/s41416-020-0756-3 (PMC7156529; doi:10.1038/s41416-020-0756-3)

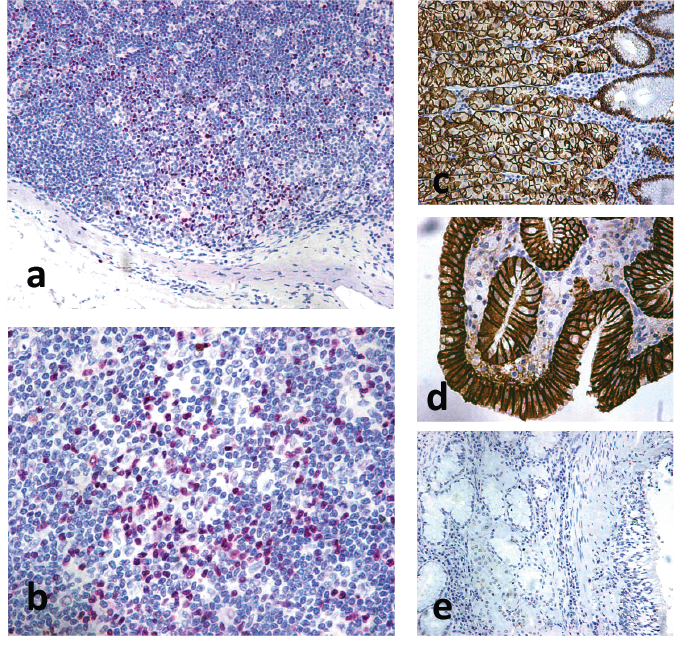

Supplement: Supplementary file 2 — Figure 1s [file 41416_2020_756_MOESM2_ESM.tif]
